# Perceptions of biodiversity loss among future decision-makers in 37 countries

**DOI:** 10.1038/s44185-024-00057-3

**Published:** 2024-08-30

**Authors:** Matthias Winfried Kleespies, Max Hahn-Klimroth, Paul Wilhelm Dierkes

**Affiliations:** Department of Bioscience Education and Zoo Biology, Goethe University Frankfurt, Frankfurt, Germany

**Keywords:** Psychology, Environmental social sciences, Social sciences

## Abstract

The decline of global biodiversity is a major environmental issue with far-reaching consequences for humans and the Earth System. When it comes to biodiversity conservation, university students play an important role because, as future decision makers, they will have an important influence on how society deals with biodiversity loss. Until now, there has been no international research examining how these future decision-makers in society perceive the causes of biodiversity loss. Using a recent method customized for this data, we show here that there are eight distinct response types across the 37 countries studied that differ in their perceptions of the drivers of biodiversity loss. In one of these response types, climate change was underestimated, while in others pollution or invasive species were rated substantially lower compared to the other main drivers. The distribution of the eight response types varied between the countries. Our results demonstrate how future decision-makers around the world evaluate the drivers of biodiversity loss. Country-specific conditions and differences between the surveyed countries were revealed. The findings serve as a starting point for decision-makers around the world to tailor education programs and policy measurements to the circumstances in their countries.

## Introduction

Due to the ongoing decline in global biodiversity, the world is facing a biodiversity crisis^1,2^. Predictions suggest that this decline will continue throughout the 21st century^3^. The current extinction rate is approximately 1000 times higher than the background rate of extinction due to human activities^4^ and may increase further in the future^5^. Biodiversity degradation has now already reached an irreversible level with unforeseeable consequences^6^. By now, it can be assumed that a major sixth mass extinction in Earth’s history is currently underway^7^.

The five main drivers of the global decline in biodiversity are well known: Habitat loss, overexploitation, pollution, climate change, and invasive species^8^. Various studies, have assigned different levels of importance to these factors^9–12^. However, ranking these drivers is criticized because it can lead to conservation actions being misguided. Therefore, it is preferable to consider the drivers collectively^13^, as they are closely interrelated and potentially reinforce each other^14,15^.

Despite the problems and the resulting severe consequences being well known, not enough actions are currently being taken to halt the loss of biodiversity^16^. The gaps in action may be due to the lack of mainstreaming of biodiversity in public policy and limited awareness of biodiversity loss among policy makers and the public^5,17^. There are also deficits in the general population’s understanding of biodiversity: studies provide evidence that many adults and high school students are not familiar with the term biodiversity^18,19^. What is understood by biodiversity often differs between individuals^20^ and the terms nature and biodiversity are often used interchangeably^21^. As a result, there is often a discrepancy between institutional definitions of biodiversity and what people understand by it^22^. These differences in perception of biodiversity can be shaped, for example, by the social or cultural group^23^.

Perceptions of environmental problems are an important factor that influences people’s behavior^24^: People are more willing to behave in a sustainable way if they perceive biodiversity loss as an imminent environmental problem^25^. Education can foster environmentally friendly behavior by increasing knowledge, perceptions, and concern about biodiversity^26–28^. Therefore, educational strategies play a crucial role, as they can make a decisive contribution to sustainability by raising awareness and knowledge about the biodiversity loss^29–31^.

Interest in investigating public perceptions of biodiversity loss has also increased in recent years. National survey instruments were developed and evaluated^32^, and there are now also approaches to studying behavioral intentions in different cultures in an international context^33^.

In the field of environmental conservation and biodiversity, higher education institutions play a crucial role: With their unbiased information, they influence the decisions of politicians and industry leaders^34^. They reach a wide audience^35^ and contribute to sustainability through research, university policy, and public engagement^36^. Training students is also a particularly important task: Universities educate the decision-makers, leaders, intellectuals, and professionals of the future^35,37,38^. While it is possible to be a decision-maker in society without a university education, universities provide critical skills and knowledge that increase the likelihood of reaching such a position^39^.

For this reason, it is particularly relevant to investigate students’ perceptions of the critical issue of biodiversity loss. The perceptions of students in the environmental field are particularly important in this context, as it is likely that they will later work in the environmental field and will therefore be confronted with environmental problems such as biodiversity loss. Since biodiversity loss is a global problem, an international perspective is also very important in this context. While student perceptions for other concepts, such as the Sustainable Development Goals^40^ or planetary boundaries^41^, have been studied in an international context, there is currently a lack of comparative studies examining how students in the environmental field worldwide assess the drivers of global biodiversity loss. Therefore, this study examines how environmental students in different countries worldwide evaluate the drivers of global biodiversity loss (main drivers) and whether they can distinguish them from drivers that have barely any impact on global biodiversity (minor drivers).

For this purpose, 4441 students in the field of environmental and sustainability studies in 37 countries were surveyed using an online questionnaire. For data analysis, a method recently developed for specifically analyzing such data sets is used^42^. It is based on unsupervised learning methods to identify patterns in the ratings, thus classifying the questionnaires of the 4441 respondents into higher-level response types. The proportion of each response type per country, referred to as the country’s ‘fingerprint’, will provide important information about the perception of the students in a country and yields to a natural measure of similarity between countries. The advantage of the methodological approach used is that the countries can be compared without requiring a structure simplification procedure (such as a PCA) beforehand^42^.

In addition to the education described at the beginning, it can also be assumed that students’ awareness of biodiversity loss may be influenced by the specific environmental challenges their country faces. To account for this, this study correlates the proportion of response types in the countries with various environmental and economic indicators that reflect a country’s biodiversity status and environmental health (CO_2_ emissions per capita, biodiversity, wealth, environmental performance, and invasive species).

We hypothesize that students from wealthier countries with higher CO_2_ emissions per capita will be less concerned about biodiversity loss and will, therefore, rate the main reasons for biodiversity loss as less severe compared to students from less wealthy countries with lower CO_2_ emissions. Similar hypotheses have previously been proposed in the literature for general environmental attitudes^43–45^. Additionally, we assume that students from countries with higher biodiversity are more likely to appreciate it^46^ and are, therefore, better able to recognize and assess the reasons for biodiversity loss. By linking the indicators with response types, we aim to understand how national contexts influence students’ perceptions of biodiversity loss.

## Results

A total of 4441 people from 37 countries were surveyed (Table 1). The analysis of the questionnaires using the data analysis method showed a separation into eight different response types, which can be clearly distinguished from each other.

**Table 1:** Sample size and mean values with standard deviation of the evaluations of the main drivers of global biodiversity loss, by country

Abbreviation	*n*	Climate Change	Exploitation	Invasive species	Habitat loss	Pollution
AUS	103	4.67 ± 0.83	4.75 ± 0.72	4.50 ± 0.71	4.80 ± 0.51	4.25 ± 0.80
BRA	96	4.92 ± 0.31	4.94 ± 0.56	4.58 ± 0.95	4.96 ± 0.54	4.63 ± 0.68
CAN	190	4.62 ± 0.99	4.81 ± 0.61	4.19 ± 1.17	4.77 ± 0.74	4.38 ± 0.85
CHN	105	4.38 ± 0.97	4.61 ± 0.78	3.94 ± 1.11	4.57 ± 0.85	4.44 ± 0.93
COL	119	4.89 ± 0.85	4.58 ± 1.18	4.23 ± 1.25	4.81 ± 0.92	4.66 ± 0.93
CRC	30	4.93 ± 0.25	4.90 ± 0.30	4.43 ± 0.92	4.90 ± 0.93	4.41 ± 1.12
DOM	112	4.84 ± 1.29	4.70 ± 1.13	4.32 ± 1.47	4.84 ± 1.19	4.54 ± 1.16
ECU	29	4.93 ± 0.25	4.86 ± 0.43	4.61 ± 1.19	4.93 ± 0.25	4.59 ± 0.93
ESP	294	4.79 ± 1.04	4.67 ± 0.93	4.27 ± 0.99	4.73 ± 0.78	4.57 ± 0.86
FRA	115	4.66 ± 1.02	4.79 ± 0.98	4.18 ± 1.20	4.80 ± 0.90	4.46 ± 0.96
GBR	97	4.76 ± 0.77	4.76 ± 0.47	3.80 ± 1.04	4.66 ± 0.69	4.34 ± 0.77
GER	340	4.43 ± 1.08	4.32 ± 1.01	4.02 ± 1.02	4.42 ± 0.90	4.26 ± 0.95
IND	57	4.77 ± 0.73	4.77 ± 0.53	4.04 ± 1.20	4.77 ± 0.84	4.23 ± 1.20
IRL	74	4.81 ± 0.68	4.82 ± 0.45	3.88 ± 1.14	4.75 ± 0.82	4.54 ± 0.66
JPN	59	4.33 ± 1.03	4.43 ± 0.97	3.86 ± 1.27	4.31 ± 0.89	4.34 ± 0.73
KEN	61	4.83 ± 0.95	4.70 ± 1.03	3.68 ± 1.72	4.86 ± 0.96	4.49 ± 1.10
KOR	48	4.56 ± 0.61	4.65 ± 0.48	4.02 ± 0.78	4.54 ± 0.61	4.4 ± 0.730
KSA	120	3.72 ± 1.78	4.56 ± 1.38	3.82 ± 1.64	4.52 ± 1.46	4.03 ± 1.65
MAR	43	4.48 ± 1.22	4.32 ± 1.51	3.56 ± 1.73	4.63 ± 1.35	4.26 ± 1.51
MEX	159	4.82 ± 0.95	4.81 ± 0.52	4.60 ± 0.84	4.83 ± 0.53	4.58 ± 0.73
NGR	85	4.60 ± 1.10	4.61 ± 1.08	3.30 ± 1.62	4.71 ± 0.89	4.52 ± 1.26
PAK	101	4.78 ± 0.78	4.55 ± 0.99	3.73 ± 1.33	4.61 ± 0.88	4.30 ± 1.13
PAN	28	4.89 ± 0.41	4.82 ± 0.54	4.64 ± 1.04	4.93 ± 0.37	5.00 ± 0.93
PER	122	4.48 ± 1.23	4.45 ± 0.86	3.99 ± 1.18	4.52 ± 0.88	4.45 ± 0.96
PHI	264	4.74 ± 0.93	4.55 ± 1.19	3.95 ± 1.54	4.66 ± 1.16	4.44 ± 1.19
POL	502	4.53 ± 1.04	4.41 ± 0.99	4.22 ± 1.09	4.45 ± 0.91	4.29 ± 0.98
POR	204	4.71 ± 0.73	4.69 ± 0.64	4.44 ± 0.94	4.78 ± 0.47	4.27 ± 0.82
PUR	56	4.89 ± 0.74	4.84 ± 0.45	3.59 ± 1.25	4.95 ± 0.29	4.66 ± 0.61
RSA	30	4.57 ± 0.62	4.77 ± 0.76	4.50 ± 0.72	4.80 ± 0.48	4.43 ± 0.76
RUS	102	3.88 ± 1.10	4.29 ± 1.12	3.96 ± 1.15	4.48 ± 0.92	4.10 ± 1.07
SGP	127	4.65 ± 0.73	4.76 ± 0.68	3.61 ± 1.23	4.70 ± 0.69	4.48 ± 0.82
SVK	131	4.53 ± 0.97	4.37 ± 1.01	3.74 ± 1.26	4.65 ± 0.82	4.32 ± 0.90
SWE	49	4.55 ± 0.67	4.94 ± 0.74	4.22 ± 1.02	4.92 ± 0.34	4.14 ± 0.95
THA	66	4.54 ± 1.08	3.70 ± 1.50	4.15 ± 1.24	4.58 ± 1.14	4.09 ± 1.32
TPE	182	4.44 ± 1.00	4.62 ± 0.91	4.02 ± 1.16	4.49 ± 0.97	4.42 ± 0.99
UAE	60	4.54 ± 0.96	4.47 ± 0.74	4.07 ± 1.11	4.75 ± 0.54	4.37 ± 0.87
USA	81	4.61 ± 0.86	4.62 ± 0.70	4.09 ± 1.00	4.53 ± 0.89	4.33 ± 0.83

*AUS* Commonwealth of Australia, *BRA* Federative Republic of Brazil, *CAN* Canada, *CHN* People’s Republic of China, *COL* Republic of Colombia, *CRC* Republic of Costa Rica, *DOM* Dominican Republic, *ECU* Republic of Ecuador, *ESP* Kingdom of Spain, *FRA* French Republic, *GBR* United Kingdom of Great Britain and Northern Ireland, *GER* Federal Republic of Germany, *IND* Republic of India, *IRL* Republic of Ireland, *JPN* Japan, *KEN* Republic of Kenya, *KOR* Republic of Korea, *KSA* Kingdom of Saudi Arabia, *MAR* Kingdom of Morocco, *MEX* United Mexican States, *NGR* Federal Republic of Nigeria, *PAK* Islamic Republic of Pakistan, *PAN* Republic of Panama, *PER* Republic of Peru, *PHI* Republic of the Philippines, *POL* Republic of Poland, *POR* Portuguese Republic, *PUR* Commonwealth of Puerto Rico, *RSA* Republic of South Africa, *RUS* Russian Federation, *SGP* Republic of Singapore, *SVK* Slovak Republic, *SWE* Kingdom of Sweden, *THA* Kingdom of Thailand, *TPE* Republic of China, *UAE* United Arab Emirates, *USA* United States of America.

**Fig. 1:**
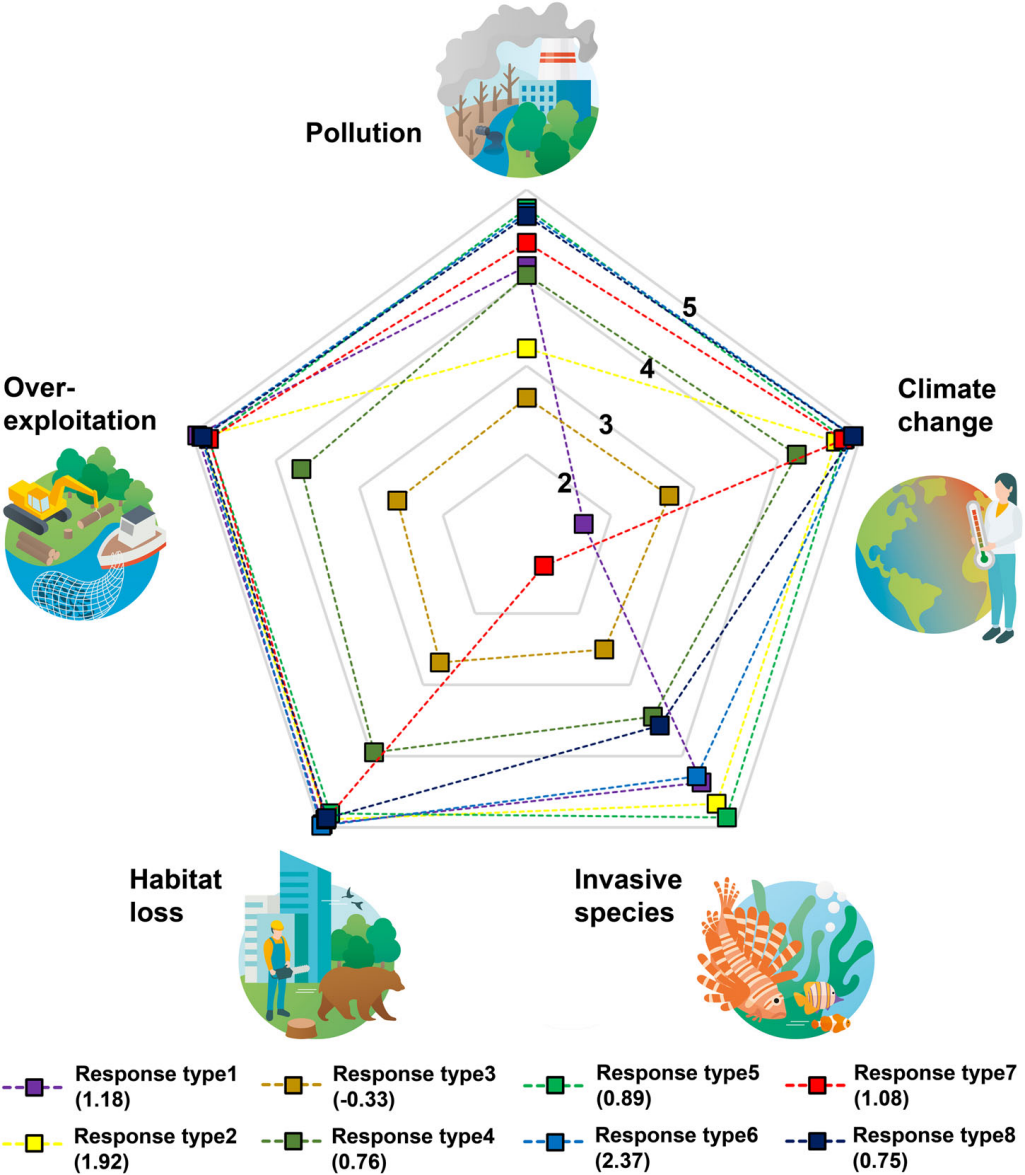
Representation of the response behavior of the eight different response types. Five indicates the assessment as a major reason. The values in the brackets show the discrimination between main and minor reasons.

In response type 1, all factors except climate change were considered to have a high influence on biodiversity decline. The minor drivers were rated as less important than the main drivers. Response type 2 shows a similar pattern, but instead of climate change, pollution was assigned a lower influence than the other main drivers. There was also good differentiation between the minor and the main drivers. In response type 3, all factors were rated as having little influence on biodiversity loss. The minor drivers were not differentiated from the main drivers in this type. In response type 4, three of the five main drivers were rated as moderately strong influencing factors (exploitation, invasive species, habitat loss), climate change and pollution were rated as slightly stronger drivers. There was a medium differentiation between main and minor drivers in this response type. In response type 5 all main drivers were considered as strong influencing factors, but the differentiation between main and minor drivers was medium. In response type 6, all main drivers were identified as very strong drivers for biodiversity loss. Invasive species, however, were assessed as slightly less important than the other main drivers. Additionally, there was a clear differentiation between minor and main drivers, as the minor drivers were rated considerably lower. The results of response type 7 and 8 were similar: The main drivers were identified as such, with the exception of invasive species: In response type 7 these were rated as a minor driver, in response type 8 as a moderate driver. In both response types, minor drivers were distinguished from main drivers (Fig. 1). The mean values and standard deviation for the main drivers and the difference between the main and minor drivers for the individual response types can be found in Table 2.

**Table 2:** Mean values and standard deviation for the five main reasons for biodiversity loss

Response type	Climate change	Exploitation	Invasive species	Habitat loss	Pollution	Discrimination
1	1.68 ± 0.82	4.94 ± 0.24	4.37 ± 1.23	4.95 ± 0.22	4.14 ± 1.19	1.18 ± 1.07
2	4.69 ± 0.57	4.88 ± 0.35	4.66 ± 0.66	4.89 ± 0.33	3.20 ± 0.91	1.92 ± 0.75
3	2.70 ± 1.37	2.55 ± 1.18	2.50 ± 1.24	2.69 ± 1.21	2.64 ± 1.31	-0.33 ± 0.9
4	4.22 ± 0.76	3.69 ± 1.00	3.44 ± 0.86	3.95 ± 0.86	4.04 ± 0.86	0.76 ± 0.77
5	4.80 ± 0.48	4.80 ± 0.49	4.86 ± 0.35	4.80 ± 0.45	4.79 ± 0.46	0.89 ± 0.70
6	4.90 ± 0.38	4.88 ± 0.35	4.28 ± 0.69	4.97 ± 0.16	4.74 ± 0.44	2.37 ± 0.80
7	4.78 ± 0.50	4.79 ± 0.46	1.33 ± 0.47	4.88 ± 0.32	4.40 ± 1.06	1.08 ± 1.04
8	4.90 ± 0.28	4.87 ± 0.31	3.57 ± 0.57	4.87 ± 0.37	4.70 ± 0.47	0.75 ± 0.51

The discrimination is the sum of the mean value of the five main reasons minus the mean value of the three minor reasons. A high positive difference value indicates that the minor reasons were rated as considerably lower influencing factors than the main reasons.

**Fig. 2:**
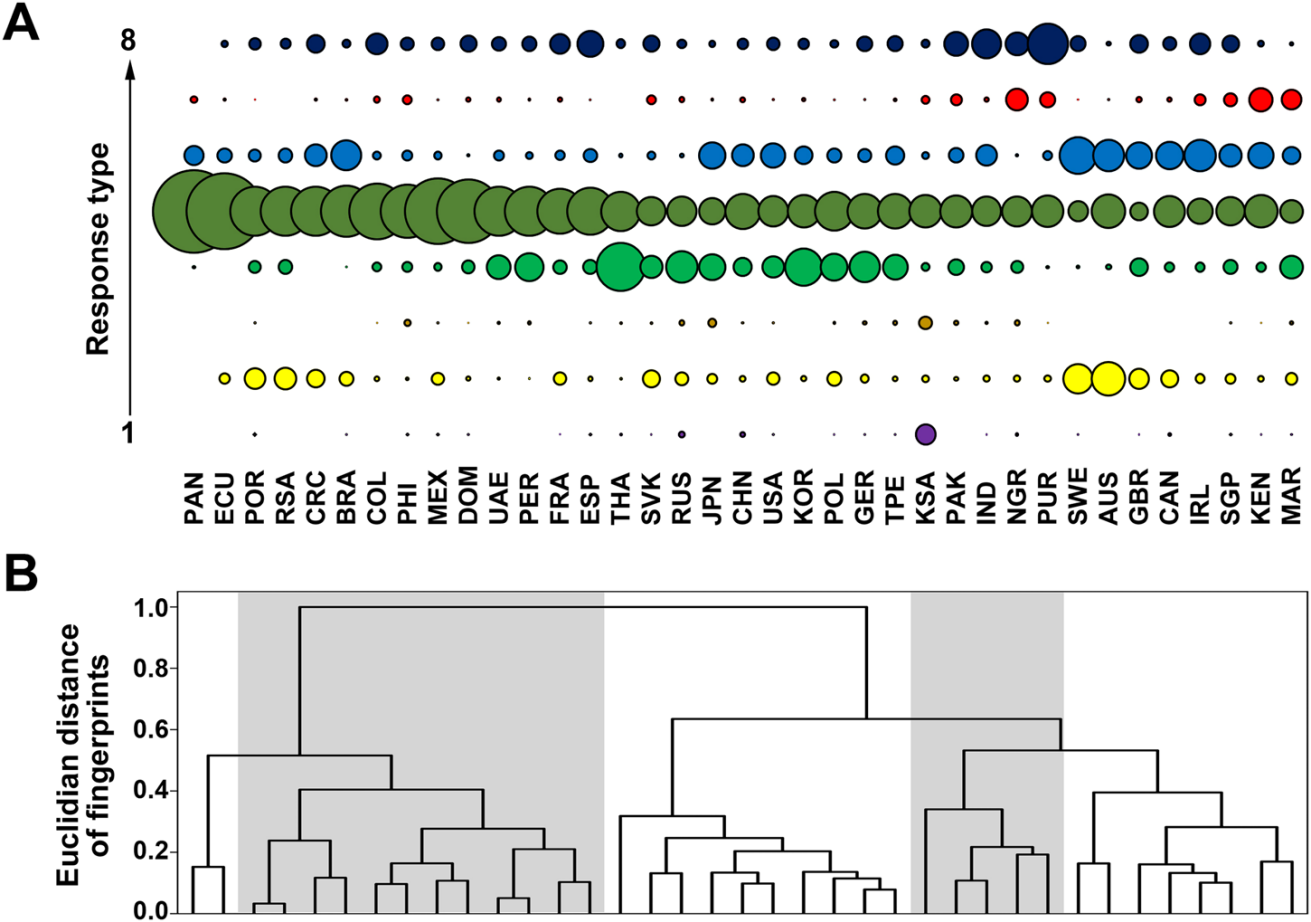
Distribution of response types within countries and similarity of countries. **A** Graphical representation of the fingerprints within each country. The larger the circle, the higher the proportion of this response type. Purple = type 1, yellow = type 2; orange = type 3; light green = type 4; dark green = type 5; blue = type 6; red = type 7; dark blue = type 8. **B** Euclidian distance between fingerprints. The country abbreviations are explained in Table 1.

The eight response types occurred in different distributions within the countries. The percentage distribution of each response type (the so-called fingerprint) for each country is shown Fig. 2A and Supplementary Table 1. An alternative visualization can be found in Supplementary Fig. 1. Using the Euclidean distance between fingerprints, similarities and differences between countries can be described (Fig. 2B). Distribution and pairwise interaction of the single questionnaire items per country can be found in Supplementary Figs. 2, 3.

To find explanations for the fingerprints of the countries, the percentages of response types within a country were correlated with country-specific indicators, using the Spearman correlation. CO_2_ (fossil CO_2_ emissions of a country) shows a medium correlation with response type 1 and 4 and a medium negative correlation with response type 7. The Environmental Performance Index (EPI) a medium negative correlation with types 3 and 7. It is also moderately correlated with response types 2 and 6. The GBI (Global Biodiversity Index) correlates moderately with type 5. NIS (Number of invasive species) shows medium correlations with types 2 and 6 and a high negative correlation with type 7. The LPI (Legatum Prosperity Index) is moderate correlated with type 2 and type 5 and moderate negatively correlated with types 3 and 7 (Table 3).

**Table 3:** Correlation and the associated p values between the distribution of the response types and the five country-specific indicators

Response type	CO_2_		EPI		GBI		NIS		LPI	
	*r*	*p*	*r*	*p*	*r*	*p*	*r*	*p*	*r*	*p*
1	**0.458**	0.005	−0.194	0.257	−0.149	0.393	0.112	0.511	−0.033	0.850
2	0.166	0.326	**0.389**	0.019	−0.306	0.074	**0.377**	0.022	**0.367**	0.028
3	0.171	0.313	**−0.445**	0.006	−0.011	0.949	−0.176	0.298	**−0.369**	0.027
4	**0.399**	0.014	−0.001	0.997	−0.155	0.373	0.089	0.599	0.121	0.482
5	−0.149	0.380	−0.180	0.292	**0.468**	0.005	−0.088	0.603	−0.323	0.055
6	0.177	0.296	**0.346**	0.039	0.012	0.944	**0.339**	0.040	**0.487**	0.003
7	**−0.402**	0.014	**−0.329**	0.050	−0.170	0.329	**−0.516**	0.001	**−0.334**	0.047
8	−0.249	0.138	0.074	0.666	−0.253	0.142	−0.060	0.723	0.101	0.557

Correlations *r* > |0.3| and *p* < 0.05 are printed in bold.

*CO*_*2*_ fussil CO_2_ emissions of a country, *EPI* Environmental Performance Index, *GBI* Global Biodiversity Index, *NIS* Number of Invasive Species, *LPI* Legatum Prosperity Index.

## Discussion

The results of our study show that there are eight different assessment patterns that differ significantly in their perception of the drivers of biodiversity loss, and that these different views are present in varying distributions in the individual countries. Respondents belonging to response type 1 strongly underestimated climate change compared to the other main drivers of global biodiversity loss. However, current research provides strong evidence that climate change is one of the main drivers of global biodiversity loss^10,11^. In addition, it can currently be assumed that in the coming years the consequences for biodiversity due to climate change will increase significantly^47^, threatening particularly areas and species that are not currently affected^48^. Therefore, it is important that especially students in the environmental field are well informed about the consequences of climate change for biodiversity. This response type occurs the least, which is probably also due to the great importance of climate change as a global environmental problem. The high correlation with the fossil CO_2_ emissions suggests that this group is particularly prevalent in regions where humans emit higher amounts of CO_2_ into the atmosphere. Especially in countries that have reached a higher percentage of type 1, additional (societal and political) measures should be taken to make future decision makers aware of the problems and consequences of climate change.

Students of response type 2 rated pollution as significantly less influential than the other main drivers. However, pollution as an environmental problem is currently more relevant than ever before, even if there is still a lack of research on the consequences of chemical pollution^49^. More than 20% of deaths and illnesses are due to some form of pollution^50^ and novel entities are already affecting the Earth system with unforeseeable consequences^51^. Especially the negative impact of pollution on biodiversity has been documented^10^. Countries with a higher proportion of this cluster should specifically educate their future decision-makers about the consequences of pollution and make them aware of the impact on global biodiversity. The correlations indicate that this type occurs more often in wealthier countries and countries that are already doing more for the health of their ecosystems. It should be noted at this point that the EPI and the wealth of a nation are highly correlated^52^. One explanation for this could be that pollution tends to play a smaller role in these countries compared to the other environmental problems and that this factor is therefore underestimated.

Response type 3 deserves attention, as the people in this group assume a low influence of all main factors and do not differentiate between main and minor drivers. While it is well known that it is sometimes difficult for students to separate main from minor drivers of biodiversity loss^53^, this group does not even perceive the main drivers as such. This is problematic because environmental behavior and concern about environmental problems are closely related: If people do not see environmental problems as such and do not worry about them, the likelihood that environmentally friendly actions will be performed decreases^54,55^. Knowledge about the existence of environmental problems is also a factor that can influence environmental behavior^56,57^.

This type is especially interesting because it directly contradicts the educational objectives of the institutions surveyed, where students of majors in the environmental field perceive the main drivers of biodiversity loss as less significant. As the response type only occurred in small numbers, it can be assumed that the curriculum or the research institutions at which the students studied were not responsible for its occurrence. Possible explanations could be for example personal or cultural influences. Cognitive biases such as optimism bias where students underestimate environmental problems could also have led to the low rating of the main drivers of biodiversity loss. Further research is needed to explain why this response type occurs.

The negative correlation with the EPI indicates that in countries that already act sustainably, this cluster occurs less frequently. The cluster also seems to occur less frequently in wealthy countries. The observation that there is a higher level of concern for environmental problems in wealthier countries has also been made in previous international studies^43,44,58^. One explanation for this is provided by Inglehart (1995), who describes that postmaterialist values lead to more positive attitudes towards environmental protection. These postmaterialist values are more common in affluent industrialized countries^45^. Therefore, there is a great need for action especially in wealthier countries and countries that have not yet focused so strongly on sustainable development. Particularly countries with a high number of response type 3 should address the concept of biodiversity loss and its drivers in more detail in university education.

Response types 4, 5, and 6 occur most frequently on average. There is a pattern of gradation between these three types: In type 4 the main drivers are rated as moderately to strongly important and there is little differentiation to the minor drivers. In type 5, all main drivers are rated as such, but there are still weaknesses in discrimination. In type 6, not only are the main drivers rated as such, but they are also very well differentiated from the minor drivers. Policy makers and decision makers in society should make their decisions based on the best evidence available^59^. Therefore, it would be desirable for all countries to increase the occurrence of type 6 through additional policy measures and the reduction of other response types, such as type 1 or 2. Since types 4 and 5 are already relatively similar to type 6, it would be desirable to promote the synthesis of types 4 to 5 to 6 through additional educational programs and outreach. Type 5 shows a medium correlation with the GBI, from which it can be concluded that, especially in countries with a high level of biodiversity, there is an increased concern for the loss of biodiversity and a wide variety of drivers are perceived as potential threat to biodiversity and assessed as main influencing factors. Type 6 shows an almost high correlation with the wealth indicator LPI. This means that this type is more likely to be found in wealthier countries. As previously explained, people from wealthy countries are more likely to show concern for environmental problems and more positive attitudes towards the environment^43,45,58^. This effect could also be a factor here.

In response types 7 and 8, all main drivers for the loss of global biodiversity are identified as such, with the exception of invasive species. It is well known that invasive species are often not recognized as a major problem^53^. Even stakeholders in contact with invasive species often have little knowledge^60^, cannot identify invasive species^61^ or tolerate their presence^62^. When invasive species are not perceived as a problem, individuals often do not advocate their management^63^. Interestingly, the occurrence of this group is closely related to the presence of invasive species in a country: the correlation shows that the number of response type 7 decreases when there are many invasive species in a country. The phenomenon that people are more likely to perceive invasive species problems when they themselves are affected is well known^64^. However, it is likely that the number of invasive species and their distribution will increase worldwide in the following years^65^. As a result, more people will come into contact with invasive species and be affected by their impact. Therefore, it is already necessary to educate about the consequences of the spread of invasive species in order to raise awareness about their dangers and possible management processes.

Since in the case of types 7 and 8 invasive species were undervalued, especially in these countries, education about invasive species and their consequences should be provided.

When interpreting the results, however, it must be noted that the selected indices can only provide initial explanations for the distribution of the response types within the countries. Studies have shown that cultural differences, social factors^66,67^ or regional factors^68^ can have a decisive impact on individual perceptions and environmental concern. In order to investigate the influence of other factors on the perception of the drivers of biodiversity loss, further studies are needed in the future in which regional comparisons within a country can also be taken into account. In particular, the influence of local biodiversity loss drivers on attitudes towards global drivers should be investigated in the future.

The distribution of response types within countries, the so-called fingerprints, provide today’s decision-makers with important information on where action is needed in each country. In general, it can be seen as very positive that across all countries, response types 5 and 6 occur most frequently, as the main drivers of global biodiversity loss are also assessed as such there. Nevertheless, the occurrence of type 6 in particular should be significantly improved in all countries, while the response types with incorrect perceptions should be reduced. In this context, the fingerprints provide the decision makers with country-specific information on which educational priorities make sense for their countries (Fig. 2B).

This study focused in particular on the perception of the global drivers of biodiversity loss. Given the interconnectedness of ecosystems and the global nature of many environmental issues, students also need a deep understanding of global biodiversity challenges. It is important that global future decision-makers are well informed about these, as knowledge and concern about environmental problems are important factors in influencing behavior and decisions^24,25,57^. However, it is important to notice that local and social factors can also play roles in influencing behaviors, and there are notable intercultural variations in the strength of these associations^67,69^.

The lack of urgent action to halt biodiversity loss is partly due to the incomplete understanding of the complex factors influencing biodiversity^70^. Policy makers are confronted with a difficult-to-structure variety of reasons for biodiversity loss, making it challenging to assess individual risks, combine them in an approach and implement a strategy to achieve sustainable development based on them. In this regard, there are current approaches to clarify these relationships in a multidimensional perspective on biodiversity to facilitate mainstreaming and support national decision-making. Soto-Navarro et al.^71^ propose a Multidimensional Biodiversity Index to link biodiversity science to the political agenda that takes into account the diversity of values underlying nature-human relationships. In this respect, it will be important to include the perceptions of environmental students as future policy makers.

The method underlying the conducted analysis was recently developed to analyze exactly such datasets: questionnaire studies in different groups such that the different groups perception of the underlying concepts might vary^42^. Moreover, the used clustering approaches combined are themselves well studied in the mathematical literature and frequently applied to understand data in different applications in sciences^72–74^. A main challenge in the analysis of questionnaires are latent group variables (as the country) which might influence the given answers. By clustering questionnaires to response types, the latent group variable country vanishes implicitly and has no influence on the response type. This means that, instead of trying to analyze a typical questionnaire given the country, a country is described by its distribution of different response types. This approach naturally allows to incorporate higher-dimensional dependencies of the single questionnaire items in different groups without the need to satisfy assumptions of classical multi variate analyses such as the Bartlet-test or the Kaiser–Meyer–Olkin test^42^.

When interpreting the different response types, it is important to note that the five main drivers of global biodiversity loss are not always weighted equally and the official rankings differ between important panels: While the IPBES classifies habitat loss as the most important driver, followed by exploitation, climate change, pollution, and invasive species, the WWF ranks invasive species in third place. According to the IUCN, most extinctions are associated with invasive species^13^. Other scientific publications give varying degrees of importance to the impact of climate change on biodiversity: Some rank it second, after habitat loss^11^, while others rank climate change fourth, ahead of invasive species^8^. The UN environment program does not rank the five reasons at all^75^. These different assessments show that ranking the reasons is not expedient when evaluating the response types, especially since biodiversity decline is usually due to several drivers and their synergies and interactions^13^. Response types that accurately identify the five major drivers of biodiversity loss and distinguish them from minor drivers may be better able to prioritize actions and allocate resources effectively to prevent biodiversity loss. On the other hand, response types that underestimate certain threats or fail to distinguish between major and minor drivers may lead to misallocation of resources and ineffective or missing conservation strategies. Therefore, when interpreting the results of this study, the focus should be on whether the main drivers were recognized as such and could be differentiated from the minor drivers. A recent study of experts (scientists in the field of biodiversity and climate change) has shown that they have a very good assessment of the causes of biodiversity loss^76^.

Biodiversity fulfils many functional and cultural roles and is therefore of particular importance - at local, national and global levels. In order to develop and implement national biodiversity conservation targets, it is important that countries tailor these targets to their own circumstances and, in doing so, train policy makers who can clearly identify drivers for biodiversity loss based on scientific facts and develop staged, target-oriented measures^5^. The aim should be that students can recognize all five main drivers of global biodiversity loss and distinguish them from non-important drivers.

The results of our study provide the first empirical evidence of how environmental students around the world assess the drivers of biodiversity loss and show a positive picture among worldwide. The five most important drivers of biodiversity loss are uniformly recognized by a high percentage (response types 5 and 6). However, the results also show potential for improvement. The results for a small proportion (response type 3) show that the students in this group assume a low influence of all main factors and do not differentiate between main and minor drivers. In other groups, individual main drivers are considered less important, which could partly be due to national circumstances. These fingerprints of individual countries are a possible starting point to determine which drivers need to be educated about in more detail. This national perspective in the individual countries is particularly important, as local conditions must be considered. However, the global perspective should also be taken into account, as the problems for biodiversity are subject to constant change and are currently increasing significantly.

Furthermore, the results show that all countries should promote response type 6 (recognition of the main drivers and differentiation from the minor drivers). In addition, the correlations of the cluster distribution with the country-specific indices provide additional information on which global factors have an influence on the students’ perceptions. The results can help to better understand how biodiversity conservation through education could be optimized globally and which scientific findings on drivers of biodiversity loss should be integrated in a more targeted way in this regard, especially in the university education of future decision makers.

Although the study was conducted with great care, some limitations must be addressed. For example, the sample size in some countries was comparatively small. This could have led to the result not being representative of the environmental students in that country.

As only students in the environmental field were surveyed in the study, the results are not representative for all students or for the population of the countries. Further studies are needed to investigate how biodiversity loss is perceived by other groups.

The study was also conducted on a voluntary basis by e-mail. It is therefore possible that people who were interested in the topic were more likely to complete the questionnaire than those who were less interested. However, as this was the case in all countries, the results remain comparable.

## Methods

### Data collection procedure

An online questionnaire was used to conduct the survey. To ensure a high level of data protection and anonymity of the participants, the survey was completed using the survey platform evasys. This platform has high standards of data and information security and is ISO-27001 certified. To collect data, scientists (professors, lecturers, laboratory directors, department heads, or other department staff) in the countries surveyed were emailed and asked to share the survey link with their students. Only scientists in the environmental field (e.g., biology, ecology and conservation, environmental science) were contacted, as their students were the target group of the study. The countries surveyed were selected by the authors with the aim of surveying a large number of diverse countries on different continents. In addition to the link to the survey, a short explanatory text describing the purpose of the study, data protection, and the voluntary nature of participation was included in the email. As the persons were informed of the voluntary nature of the study and no data was requested that could enable the person to be identified, written consent was not obtained. After being informed of the voluntary nature of the study, participation was considered as informed consent. The survey was conducted in one of the official languages of the countries surveyed. The translations were carried out beforehand by native speakers and checked by another person. Data from students who stated in the questionnaire that they were not majoring in the environmental field, for example because they were taking a surveyed course as part of another degree program, were excluded from the analysis. Data from PhD or students exchange students who came from another country were also not included in the data analysis. The sample size per country is shown in Supplementary Table 2. The minimum sample size was set at 25 students per country. Countries with a smaller sample were not used in the analysis. The survey took place between September 2020 and July 2021 and was approved by the ethics committee of the science didactic institutes and departments of the Goethe University Frankfurt am Main under approval number 15-WLSD-2104. If universities in which the survey was conducted required the additional approval of a local ethics committee, this was also obtained.

### Measuring instrument

The battery of questions used for this study began with a brief definition of the term biodiversity: “Biodiversity (the diversity of species, the diversity of ecosystems, genetic diversity) is today undergoing massive global change. Please assess the extent to which the following reasons are responsible for the decline in global biodiversity.” The students were asked in closed-ended items to rate on a 5 points Likert-scale (minor impact to major impact) how much impact they thought the following drivers had on the decline of global biodiversity. Nine possible drivers for biodiversity loss were presented to the students. Among these were the five main drivers of global biodiversity decline (habitat loss, overexploitation, pollution, climate change, and invasive species) and four minor drivers that do not have a significant impact on global biodiversity (electromagnetic pollution, entering nature reserves, factory and vehicle noise and the internet). These minor drivers were chosen by the authors with the aim of selecting concepts that may sound plausible, but objectively have no or a negligible impact on global biodiversity. These minor drivers were used to investigate whether students can differentiate between significant global drivers of biodiversity loss and drivers that have no (global) impact on biodiversity loss. The goal was to determine whether the students really have an understanding of the drivers of biodiversity loss or whether everything is assessed as a problem without any reflection. Due to content-related concerns, the minor driver “entering nature reserves” was not included in the analysis and work continued with only 3 minor drivers. As these minor drivers equally have no significant effect on global biodiversity, their mean value was used for the analysis. In addition, demographic data such as age, gender, semester, university, and country were collected.

### Methodological procedure

The analysis of the given dataset is, actually, a typical example of a “supervised learning task”. Each questionnaire consists of a number of variables, called “features”, and the dependent variable, the country, is known. In machine learning language, the country would be called “label”. Supervised learning means that a model needs to be found that enables us to predict the country from the features, thus the given answers. If a decent model is found, this indicates that the country can be estimated from the answers given in a questionnaire and conclusions can then be drawn by understanding the importance of single features or their influence on the label. But this approach cannot be applied to most of studies based on questionnaires. Indeed, as a relatively small number of possible answers can be given, the same features will lead to different labels quite frequently - this is a contradiction to very basic assumptions in supervised learning.

“Unsupervised learning tasks”, on the other hand, are designed to find patterns in a dataset, or to exploratively explain a dataset where labels are not present (or not known). Of course, standard tools from unsupervised learning theory can be applied to a collection of questionnaires, and questionnaires can be grouped (or “clustered”) by such algorithms. However, this does not allow to use the actual underlying information that different questionnaires stem from different countries. Recently, a methodological approach combining classical statistics and unsupervised learning was published in the data scientific community^42^. This method uses unsupervised learning techniques in the first step to cluster questionnaires. Second, for each country, a “fingerprint” is calculated which encodes the proportion of questionnaires of every cluster in the country. Third, unsupervised learning on those fingerprints is used to measure similarity between different clusters and classical statistical tools are applied. While a method paper appeared recently, we are not aware of any study in the field of environmental psychology that already applies the method to a cross-country study, and we believe that this approach itself is of interest to a larger community.

### Analysis

The data were processed and analyzed as proposed by ref. ^42^. As previously described, there are 8 items represented by integers between 1 (low impact on global biodiversity) and 5 (high impact on global biodiversity) in each questionnaire. To keep the sample size as large as possible, incomplete questionnaires were imputed using scikit ‘s KNN imputation function. More specifically, the missing values of each sample were imputed by the average of the 8 nearest neighbors, where the closeness of two questionnaires was measured only by the features that neither was missing. This is a standard approach to impute data reliably^77^. In the second step, a feature engineering step, the 3 minor drivers were replaced by the difference between the means of the main and minor drivers, respectively. This adds an integer coordinate between −5 and 5 to each questionnaire, representing discriminability. Thus, each questionnaire is now represented by a 6th dimensional vector. Third, the data were slightly perturbed by Gaussian noise with mean zero and variance 0.001, independently in each coordinate, to ensure that there are no duplicates, which is a requirement for numerical stability of the clustering algorithms, while keeping each questionnaire in the dataset. In addition, this perturbation increases the stability of the final clustering against small changes in the original data, which is a desirable property^78^. The fourth step was to cluster the questionnaires according to their similarity. As the clustering algorithm, Ward’s clustering algorithm was chosen as suggested by ref. ^42^. The algorithm was implemented by Python’s scikit library and identified eight clusters of questionnaires. Following the notation of the method paper, the central element (“the average questionnaire”) is called “response type”. More specifically, the algorithm takes the number of clusters as input, and the number of clusters was optimized for stability such that fewer clusters show significantly more variance within a cluster, but an additional cluster does not noticeably reduce variance.

One decision during the analysis is to determine the number of response types. Hahn-Klimroth et al.^42^ suppose to use the “gap statistic” to determine the number of response types^42^. The main idea behind the gap statistic is to compare the given data to “randomly generated data without any structure”. Given a number of clusters, the corresponding gap value signifies how unlikely it is to find the cluster structure on the random data. Hence, the optimal number of clusters corresponds to either a local maximum or at least an “elbow” in the scree-plot which plots the gap value against the number of clusters. In the current study, the optimal number of clusters turns out to be 8.

Given the eight response types, a so-called “fingerprint” was calculated for each country as an 8-dimensional point such that the i-th coordinate represents the proportion of questionnaires of type i in the country. These fingerprints come with a natural interpretation of how similar two countries are, namely when their Euclidean distance is small. This similarity can be expressed visually as a “dendrogram”, sometimes called a “phylogenetic tree”. Again, the dendrogram connections are defined by Ward’s method. With the fingerprints, it is not only possible to measure the similarity between countries, but also to apply standard regression tools such as Spearman’s rank correlation, implemented in Python’s statistics library. More precisely, it is possible to measure the correlation between the proportion of type i questionnaires in a country and known indices. Here we call a correlation coefficient | r | > 0.3 a moderate correlation and say that the correlation is significantly different from zero if the corresponding *p* value is at most 0.05.

### Indices

Five indices were selected to be used to explain the distribution of the types of questionnaires within different countries.

1. Fossil CO_2_ emissions [CO_2_] of a country from 2021: Countries’ CO_2_ emissions from fossil emissions. This includes fossil fuel combustion, industrial processes and product use^79^.
2. Environmental Performance Index from 2022 [EPI]: This is an index that examines how environmentally sustainable a country is using 40 performance indicators^80^.
3. Legatum Prosperity Index from 2021 [LPI]: With a total of 300 individual indicators from 12 subcategories, the LPI evaluates the prosperity of a county^81^.
4. Global Biodiversity Index [GBI] from 2022: This index takes into account the diversity of bird, amphibian, fish, mammal, reptile and plant species in a country^82^.
5. Number of invasive species [NIS]: Number of reported invasive species in a country^83^.

### Supplementary information

Supplementary information

## Data Availability

The datasets generated during and analysed during the current study are available from the corresponding author on reasonable request.
