# Supplementary material for: Perceptions of biodiversity loss among future decision-makers in 37 countries
Source: NPJ Biodivers. 2024 Aug 30;3:21. doi: 10.1038/s44185-024-00057-3 (PMC11364627; doi:10.1038/s44185-024-00057-3)
Supplement: Supplementary file 1 — Supplementary information [file 44185_2024_57_MOESM1_ESM.pdf]

Supplementary Information

Supplementary Table 1: Fingerprints of the individual countries (percentage distribution of the response types (1-8) within the surveyed countries) rounded to two decimal points.

Country	1	2	3	4	5	6	7	8
AUS	0.00%	30.10%	0.00%	5.83%	30.10%	28.16%	0.97%	4.85%
BRA	1.04%	13.54%	0.00%	2.08%	44.79%	27.08%	3.13%	8.33%
CAN	3.68%	16.32%	0.00%	9.47%	27.89%	24.74%	5.26%	12.63%
CHN	5.71%	6.67%	2.86%	17.14%	31.43%	20.00%	5.71%	10.48%
COL	0.00%	5.88%	1.68%	9.24%	48.74%	8.40%	6.72%	19.33%
CRC	0.00%	16.67%	0.00%	0.00%	43.33%	20.00%	3.33%	16.67%
DOM	0.89%	5.36%	1.79%	12.50%	55.36%	3.57%	5.36%	15.18%
ECU	0.00%	10.34%	0.00%	0.00%	65.52%	13.79%	3.45%	6.90%
ESP	0.68%	5.44%	1.02%	13.61%	41.16%	12.93%	1.36%	23.81%
FRA	1.74%	12.17%	0.00%	13.04%	40.00%	9.57%	5.22%	18.26%
GBR	2.06%	18.56%	0.00%	16.49%	16.49%	23.71%	6.19%	16.49%
GER	1.18%	8.53%	4.71%	27.35%	30.59%	12.35%	1.18%	14.12%
IND	1.75%	7.02%	3.51%	10.53%	26.32%	19.30%	5.26%	26.32%
IRL	0.00%	9.46%	0.00%	9.46%	22.97%	28.38%	10.81%	18.92%
JPN	0.00%	10.17%	8.47%	23.73%	23.73%	23.73%	3.39%	6.78%
KEN	1.64%	6.56%	1.64%	9.84%	29.51%	22.95%	21.31%	6.56%
KOR	0.00%	6.25%	0.00%	33.33%	29.17%	16.67%	4.17%	10.42%
KSA	18.33%	7.50%	12.50%	8.33%	29.17%	7.50%	8.33%	8.33%
MAR	2.33%	11.63%	4.65%	20.93%	20.93%	16.28%	18.60%	4.65%
MEX	0.63%	11.95%	0.63%	8.18%	57.23%	8.18%	1.26%	11.95%
NGR	3.53%	7.06%	5.88%	11.76%	27.06%	3.53%	20.00%	21.18%
PAK	0.00%	4.95%	4.95%	14.85%	28.71%	13.86%	10.89%	21.78%
PAN	0.00%	0.00%	0.00%	3.57%	71.43%	17.86%	7.14%	0.00%
PER	0.00%	2.46%	4.10%	25.41%	43.44%	7.38%	3.28%	13.93%
PHI	0.76%	3.79%	7.20%	9.85%	46.97%	9.85%	9.09%	12.50%
POL	1.59%	13.35%	3.19%	24.30%	34.66%	13.35%	1.39%	8.17%
POR	0.49%	19.12%	0.98%	11.76%	43.14%	11.76%	1.47%	11.27%
PUR	0.00%	7.14%	1.79%	3.57%	28.57%	8.93%	14.29%	35.71%
RSA	0.00%	20.00%	0.00%	13.33%	43.33%	13.33%	0.00%	10.00%
RUS	6.86%	12.75%	5.88%	28.43%	26.47%	4.90%	5.88%	8.82%
SGP	0.79%	10.24%	0.79%	13.39%	25.98%	20.47%	12.60%	15.75%
SVK	1.53%	16.03%	3.05%	20.61%	25.95%	8.40%	9.16%	15.27%
SWE	2.04%	26.53%	0.00%	4.08%	18.37%	32.65%	2.04%	14.29%
THA	3.03%	3.03%	3.03%	42.42%	34.85%	4.55%	0.00%	9.09%
TPE	1.10%	5.49%	4.95%	22.53%	30.77%	17.03%	3.85%	14.29%
UAE	0.00%	3.33%	3.33%	21.67%	43.33%	10.00%	5.00%	13.33%
USA	1.23%	12.35%	2.47%	19.75%	27.16%	22.22%	2.47%	12.35%

Supplementary Table 2:

Abbreviation	n	Male	Female	Diverse	No answer
AUS	103	38	61	3	1
BRA	96	33	61	1	1
CAN	190	50	131	6	3
CHN	105	36	68	1	0
COL	119	49	69	0	1
CRC	30	11	19	0	0
DOM	112	36	76	0	0
ECU	29	12	17	0	0
ESP	294	101	187	3	3
FRA	115	51	63	0	1
GBR	97	34	58	5	0
GER	340	95	236	3	6
IND	57	20	36	0	1
IRL	74	26	46	2	0
JPN	59	26	32	0	1
KEN	61	36	23	0	2
KOR	48	20	26	2	0
KSA	120	118	2	0	0
MAR	43	11	32	0	0
MEX	159	72	85	0	2
NGR	85	47	38	0	0
PAK	101	24	77	0	0
PAN	28	8	20	0	0
PER	122	54	65	0	3
PHI	264	106	157	0	1
POL	502	125	372	4	1
POR	204	91	110	1	2
PUR	56	11	45	0	0
RSA	30	10	20	0	0
RUS	102	26	74	1	1
SGP	127	49	74	1	3
SVK	131	33	96	1	1
SWE	49	12	36	1	0
THA	66	15	51	0	0
TPE	182	101	81	0	0
UAE	60	60	0	0	0
USA	81	31	48	2	0

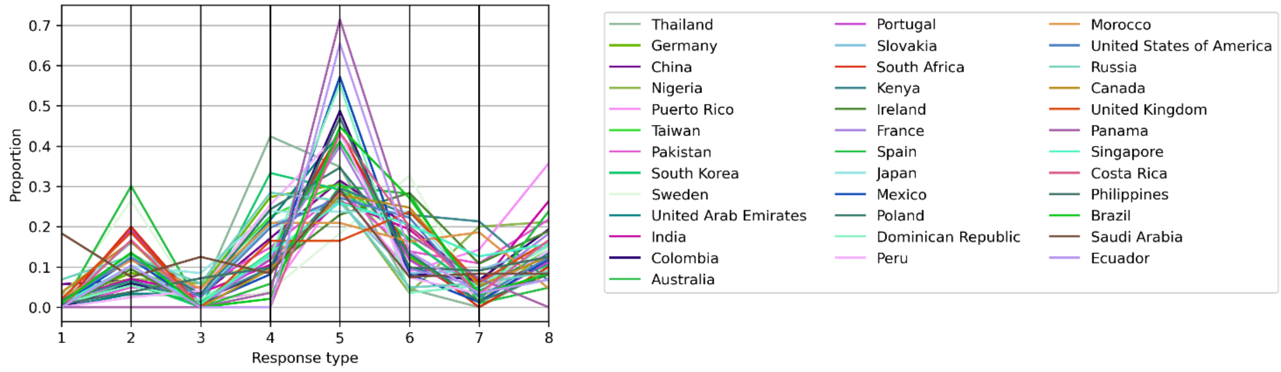

Supplementary Figure 1: Fingerprints of the individual countries (response type 1-8) as a line chart.

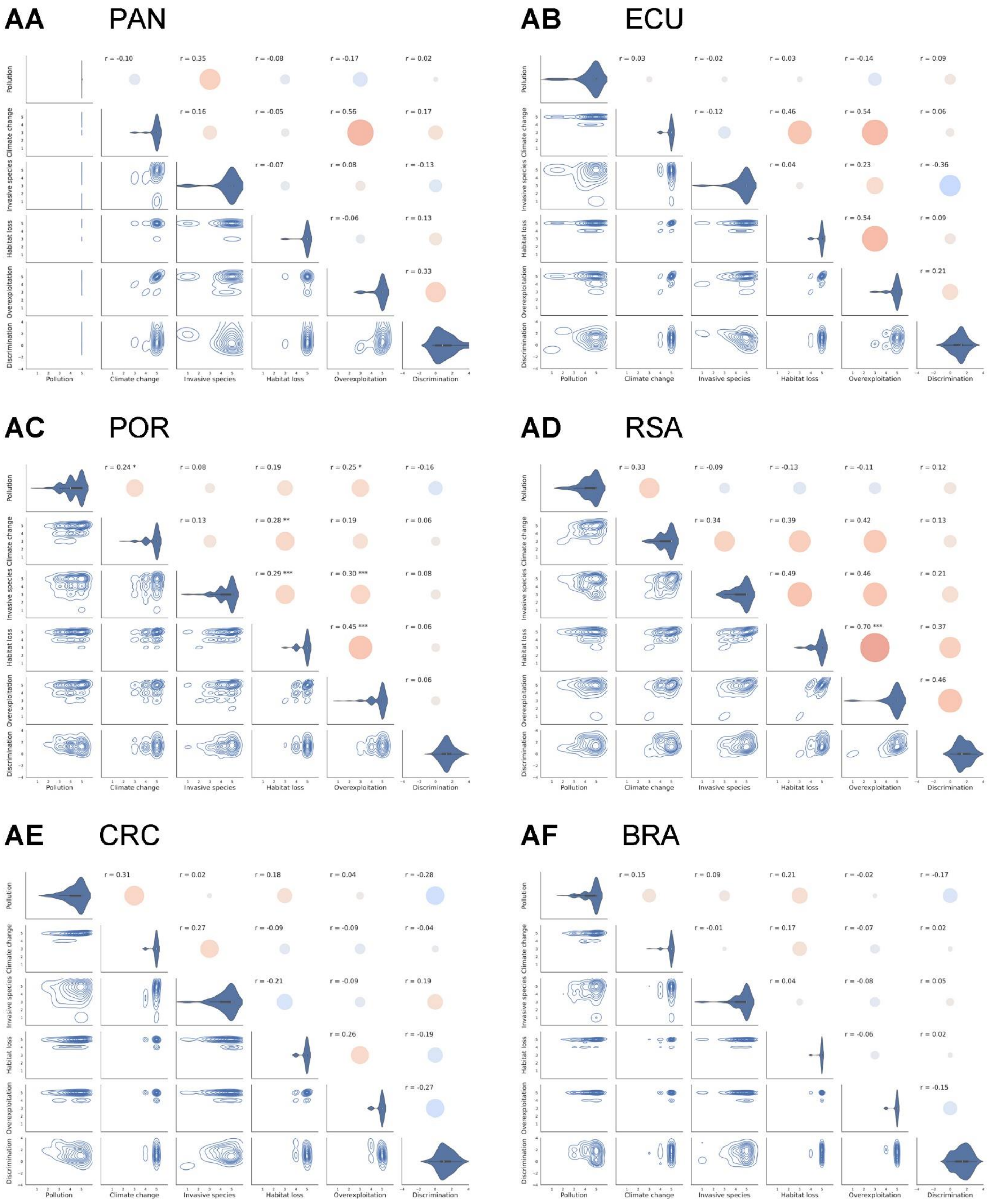
AA PAN
r = -0.10
r = 0.35
r = -0.08
r = -0.17
r = 0.02
r = 0.16
r = -0.05
r = 0.56
r = 0.17
r = -0.07
r = 0.08
r = -0.13
r = -0.06
r = 0.13
r = 0.33
AB ECU
r = 0.03
r = -0.02
r = 0.03
r = -0.14
r = 0.09
r = -0.12
r = 0.46
r = 0.54
r = 0.06
r = 0.04
r = 0.23
r = -0.36
r = 0.54
r = 0.09
r = 0.21
AC POR
r = 0.24 *
r = 0.08
r = 0.19
r = 0.25 *
r = -0.16
r = 0.13
r = 0.28 **
r = 0.19
r = 0.06
r = 0.29 ***
r = 0.30 ***
r = 0.08
r = 0.45 ***
r = 0.06
r = 0.06
AD RSA
r = 0.33
r = -0.09
r = -0.13
r = -0.11
r = 0.12
r = 0.34
r = 0.39
r = 0.42
r = 0.13
r = 0.49
r = 0.46
r = 0.21
r = 0.70 ***
r = 0.37
r = 0.46
AE CRC
r = 0.31
r = 0.02
r = 0.18
r = 0.04
r = -0.28
r = 0.27
r = -0.09
r = -0.09
r = -0.04
r = -0.21
r = -0.09
r = 0.19
r = 0.26
r = -0.19
r = -0.27
AF BRA
r = 0.15
r = 0.09
r = 0.21
r = -0.02
r = -0.17
r = -0.01
r = 0.17
r = -0.07
r = 0.02
r = 0.04
r = -0.08
r = 0.05
r = -0.06
r = 0.02
r = -0.15
Pollution
Climate change
Invasive species
Habitat loss
Overexploitation
Discrimination

AG COL

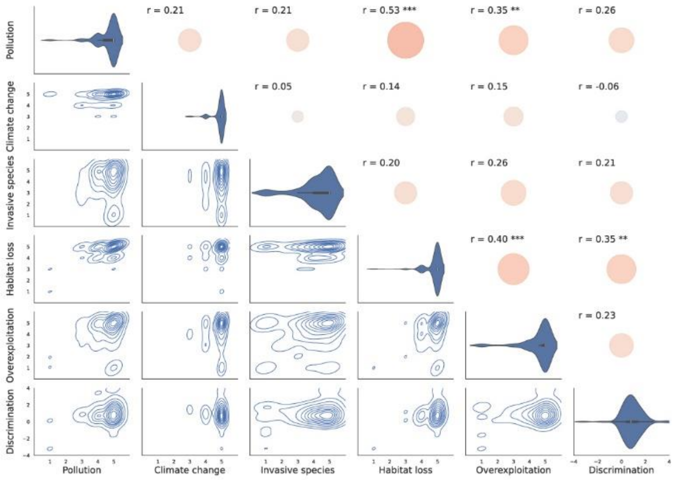

AH PHI

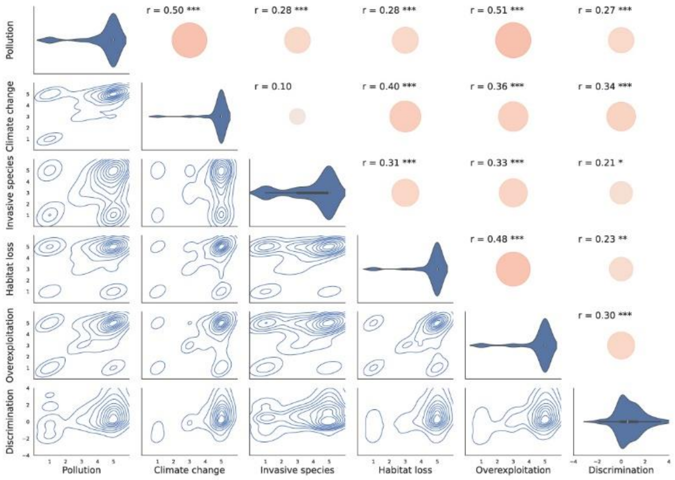

AI MEX

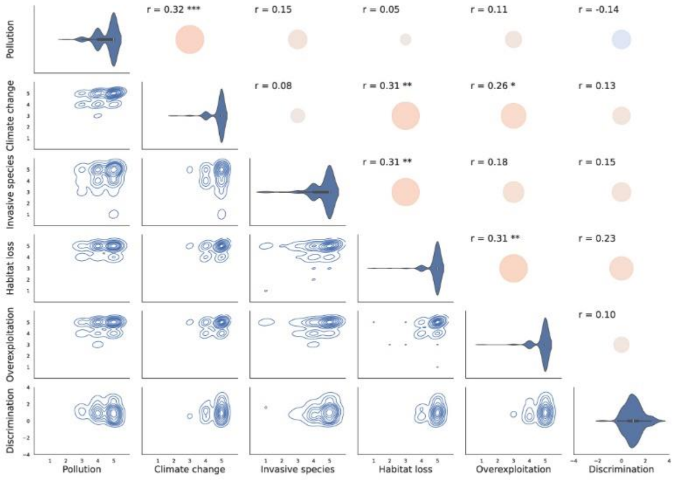

AJ DOM

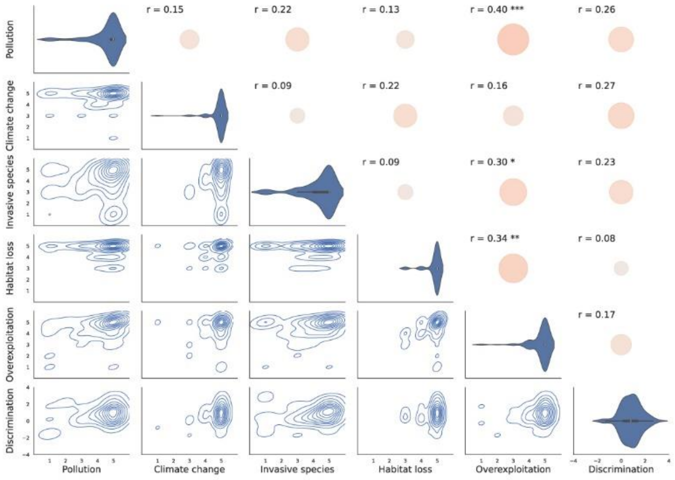

AK UAE

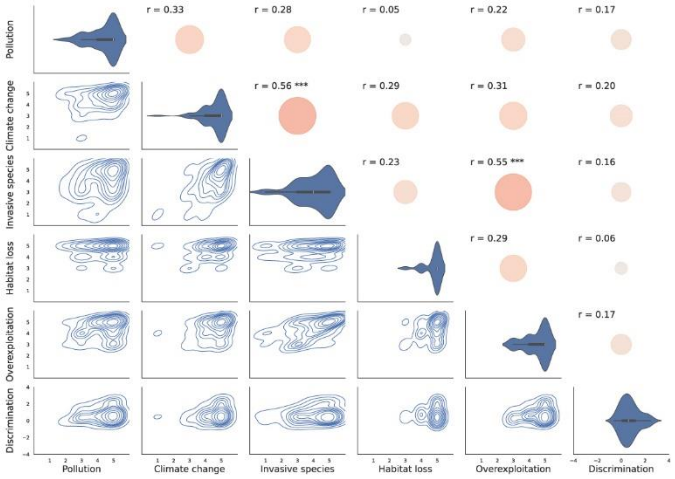

AL PER

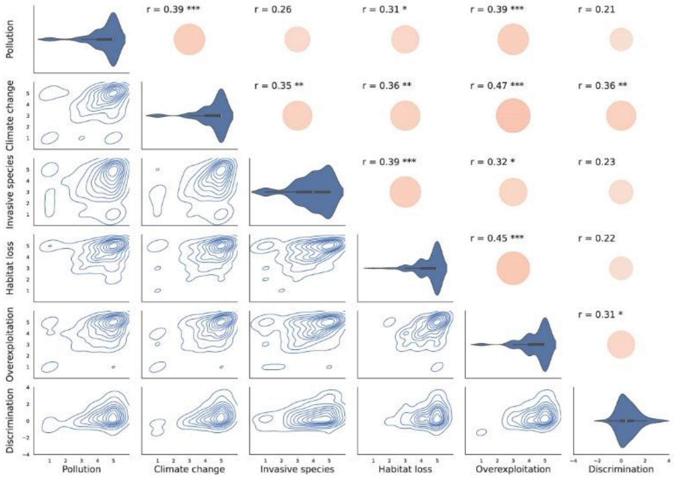

AM FRA

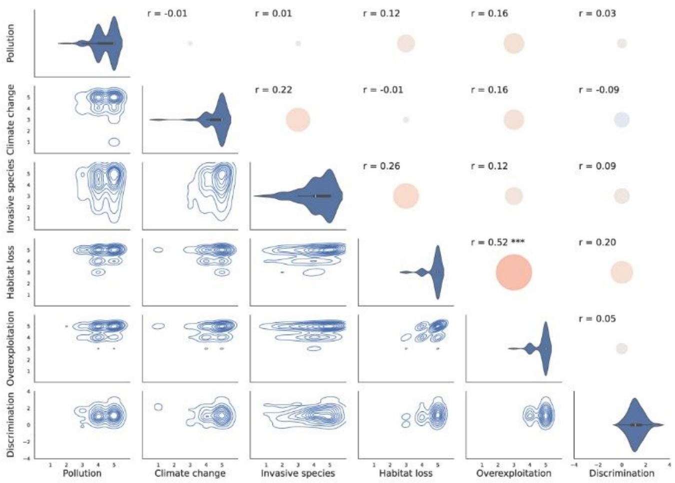

AN ESP

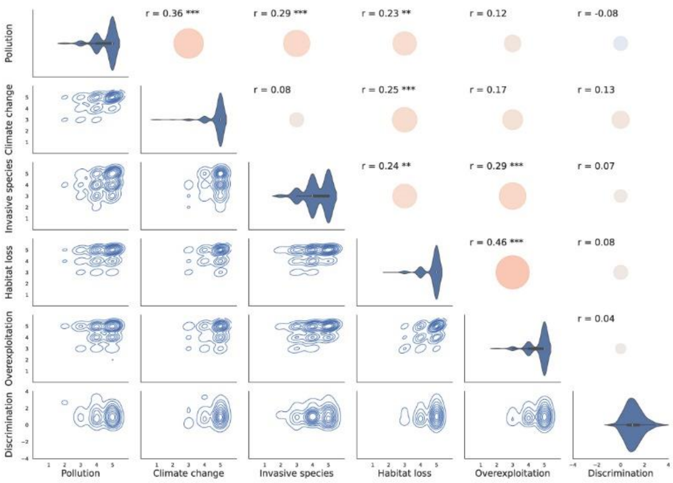

AO THA

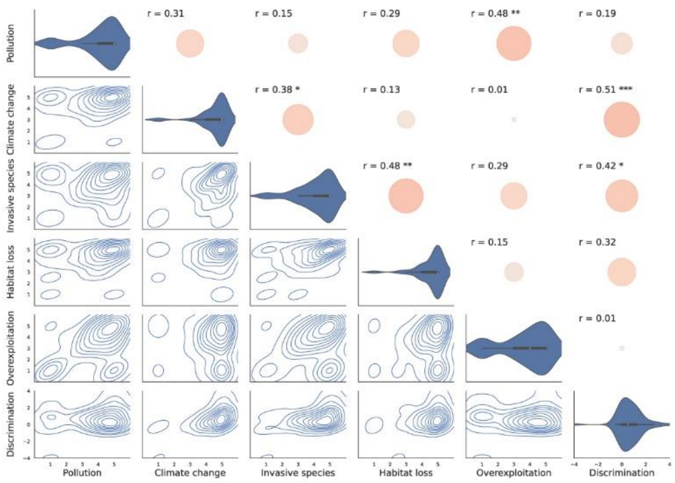

AP SVK

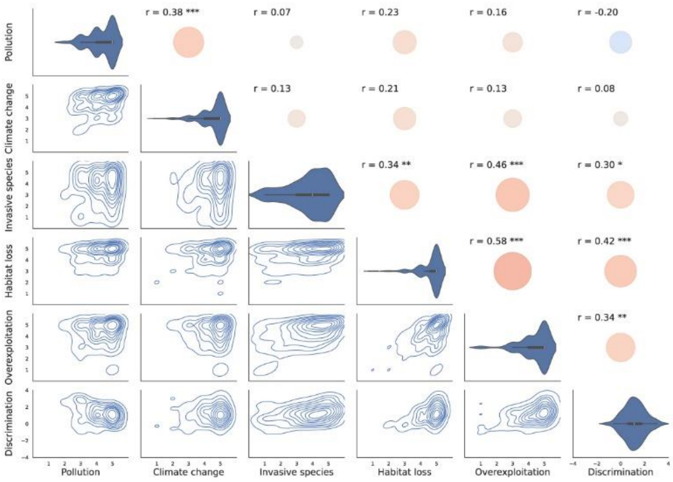

AQ RUS

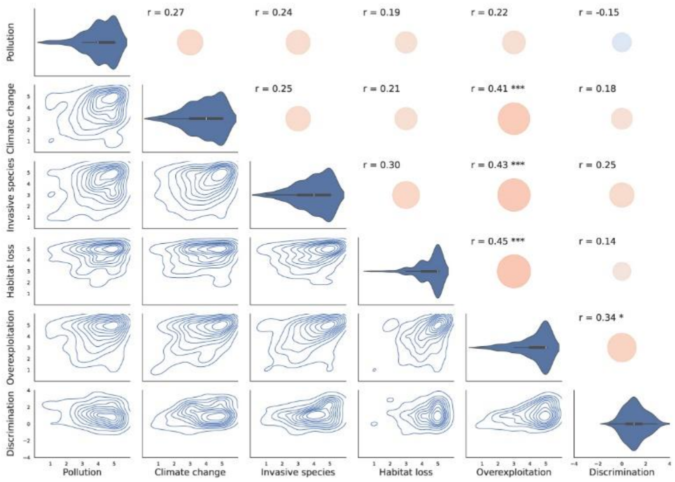

AR JPN

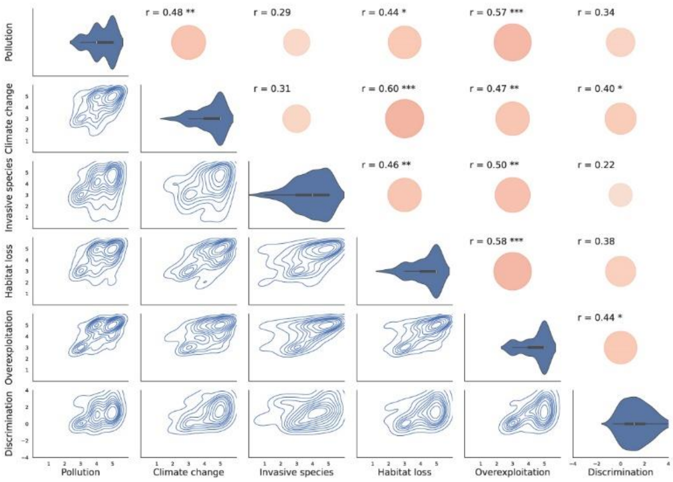

AS CHN

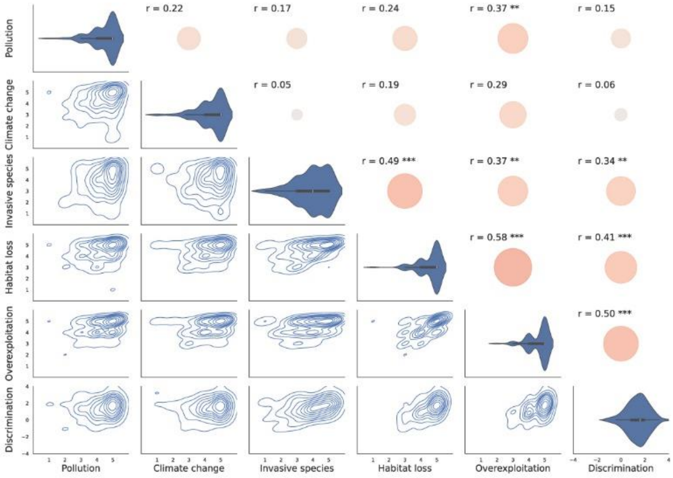

AT USA

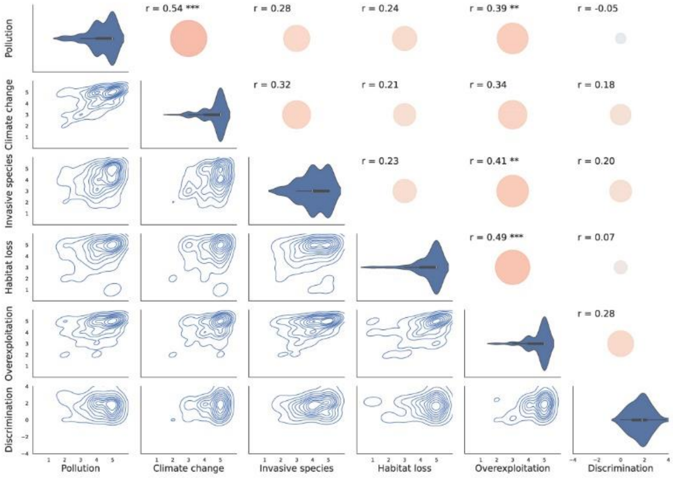

AU KOR

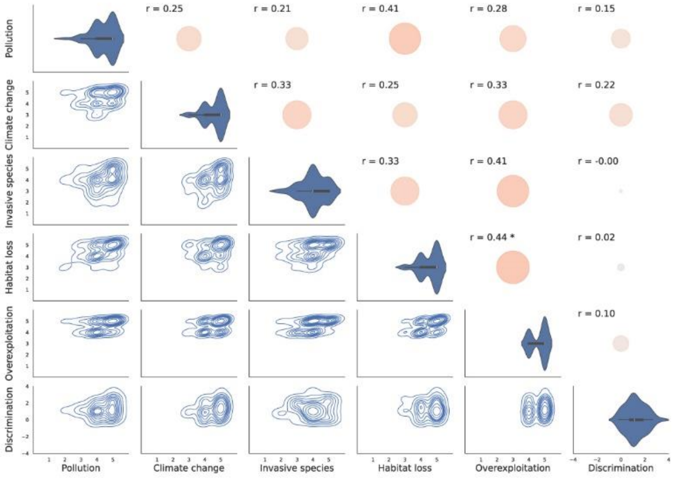

AV POL

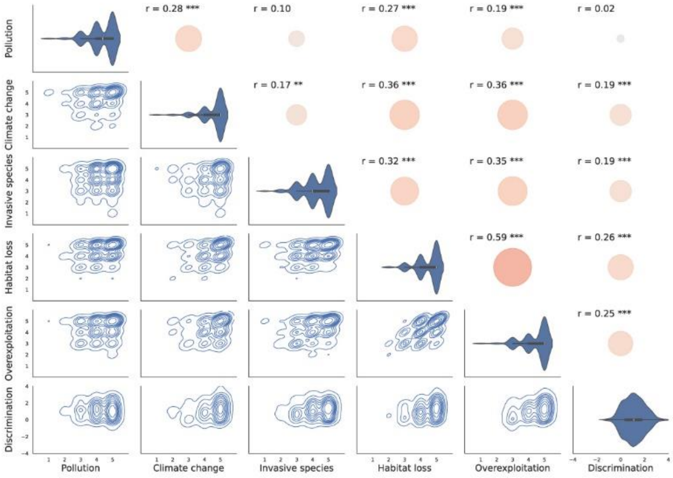

AW GER

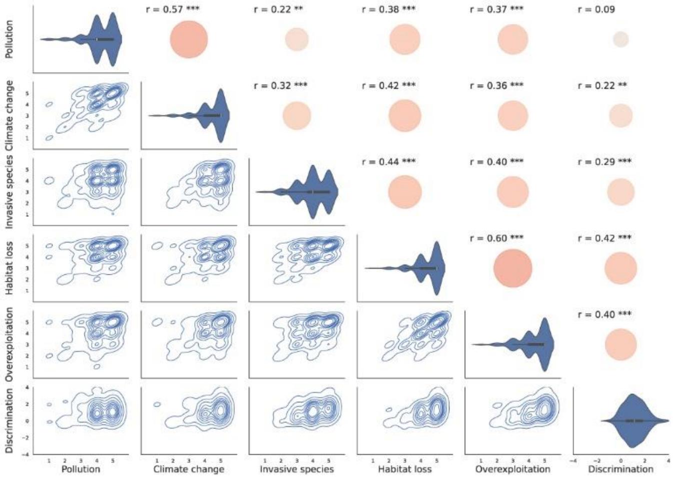

AX TPE

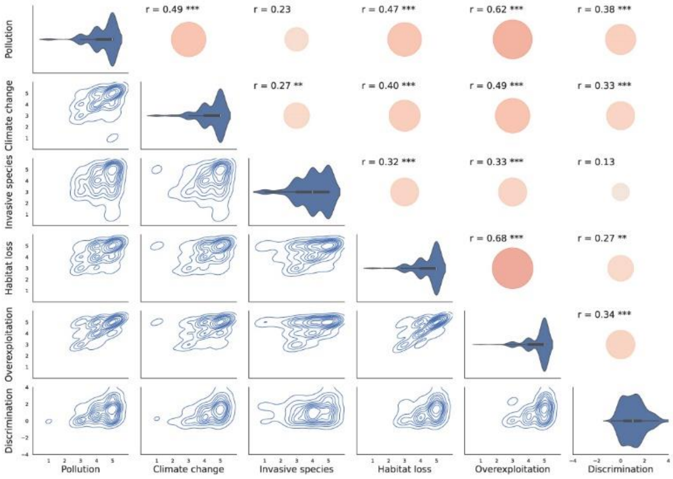

AY KSA

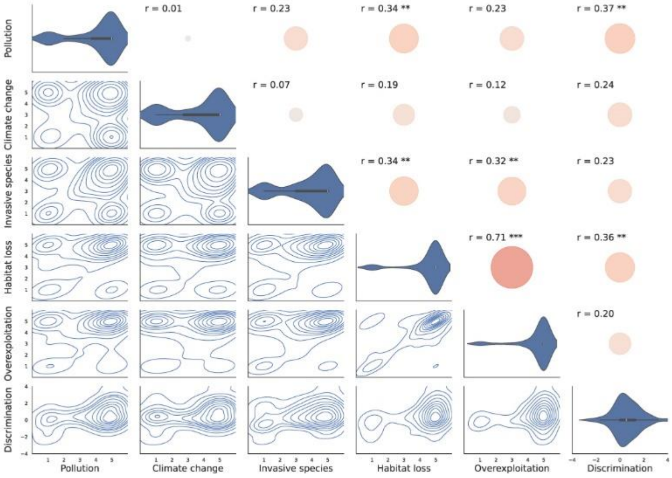

AZ PAK

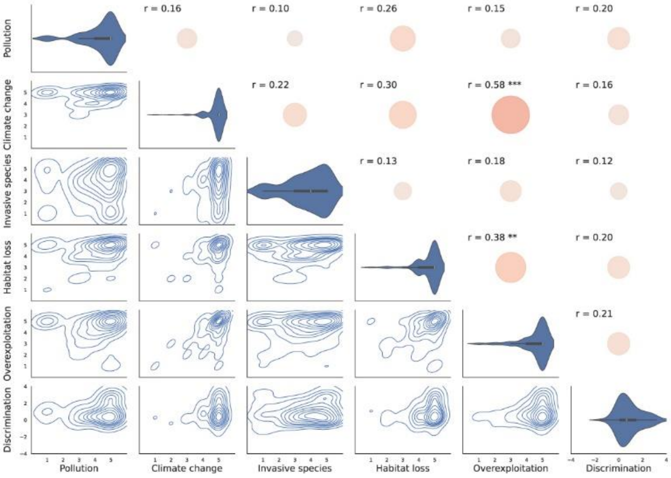

BA IND

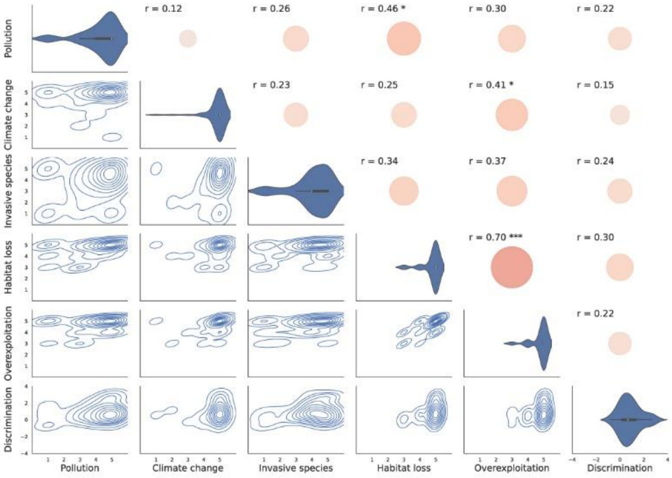

BB NGR

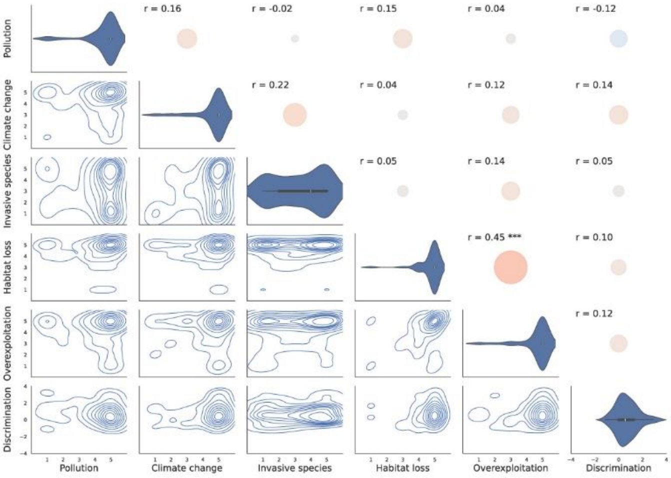

BC PUR

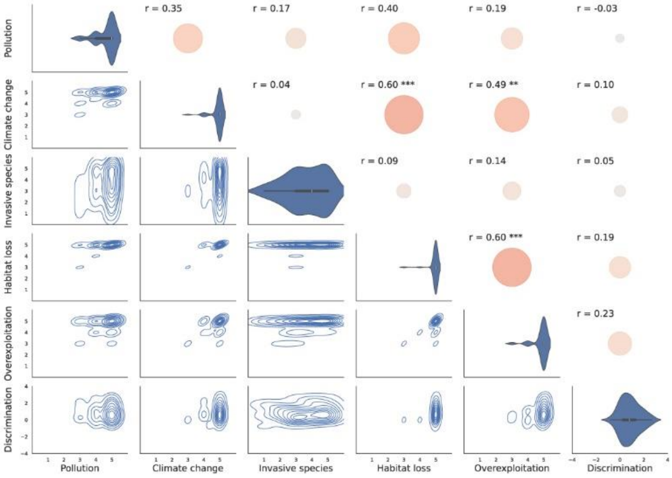

BD SWE

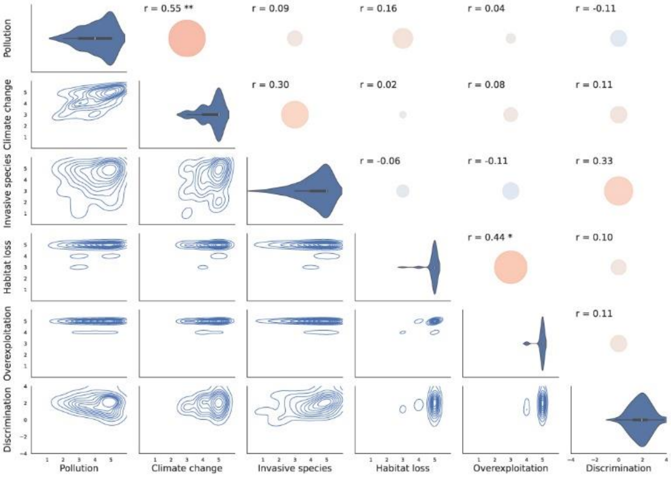

BE AUS

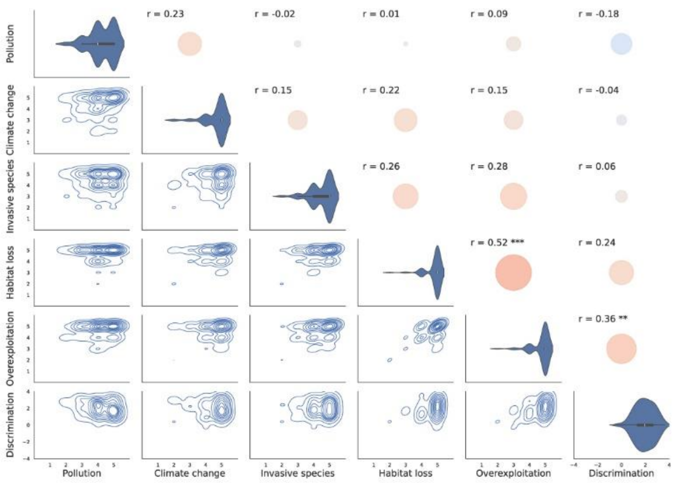

BF GBR

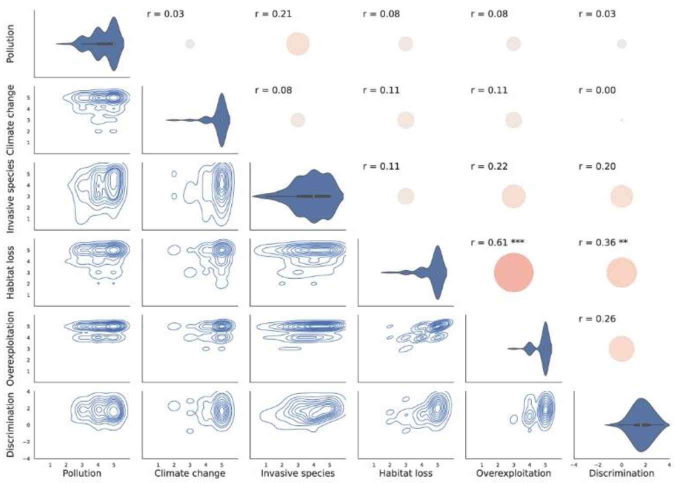

BG CAN

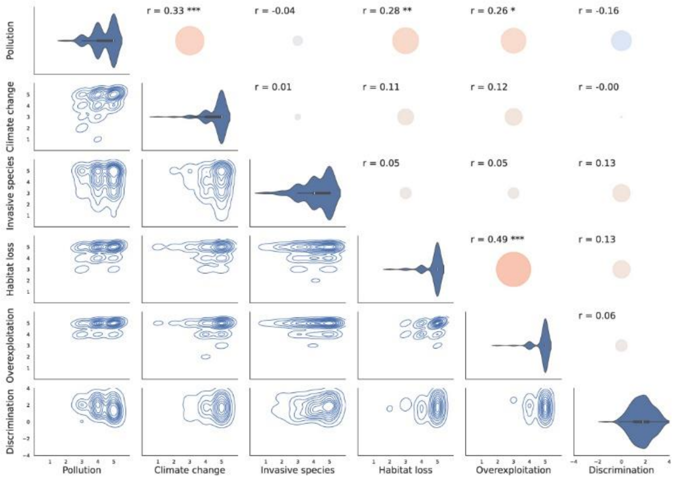

BH IRL

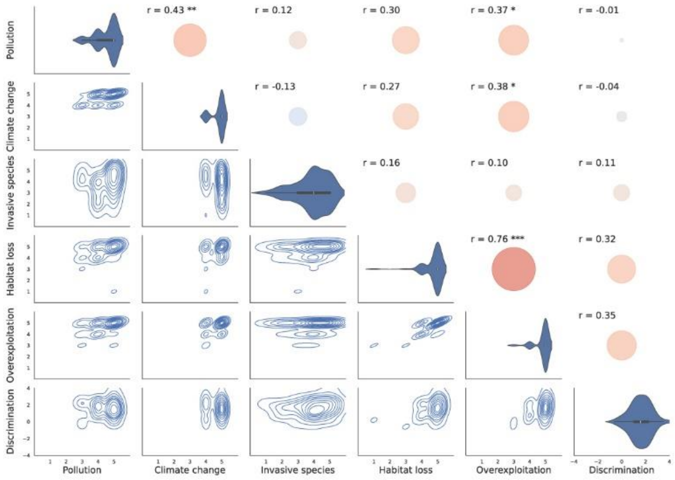

BI SGP

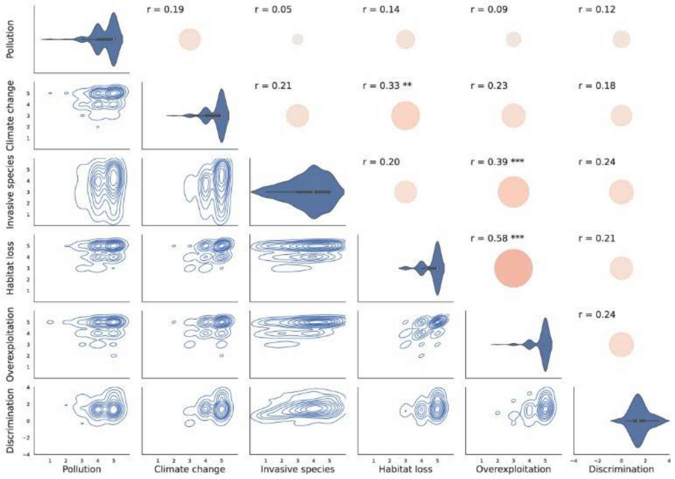

BJ KEN

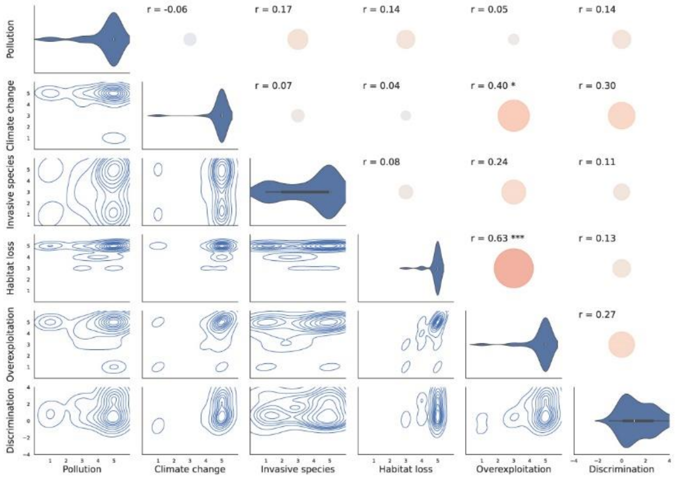

BK MAR

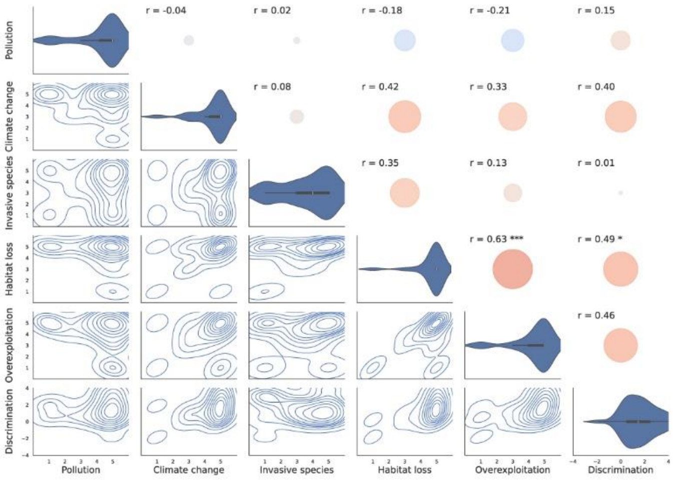

Supplementary Figure 2: Distribution and pairwise interaction of the single questionnaire items per country. The diagonal shows a violin plot while the KDE plots below the diagonal allow to visualize the joint distribution of two items each. The upper diagonal contains the correlations between the features. The order of the countries results from the Euclidian distance of the fingerprints. AA: Republic of Panama; AB: Republic of Ecuador; AC: Portuguese Republic; AD: Republic of South Africa; AE: Republic of Costa Rica; AF: Federative Republic of Brazil; AG: Republic of Colombia; AH: Republic of the Philippines; AI: United Mexican States; AJ: Dominican Republic; AK: United Arab Emirates; AL: Republic of Peru; AM: French Republic; AN: Kingdom of Spain; AO: Kingdom of Thailand; AP: Slovak Republic; AQ: Russian Federation; AR: Japan; AS: People's Republic of China; AT: United States of America; AU: Republic of Korea; AV: Republic of Poland; AW: Federal Republic of Germany; AX: Republic of China; AY: Kingdom of Saudi Arabia; AZ: Islamic Republic of Pakistan; BA: Republic of India; BB: Federal Republic of Nigeria; BC: Commonwealth of Puerto Rico; BD: Kingdom of Sweden; BE: Commonwealth of Australia; BF: United Kingdom of Great Britain and Northern Ireland; BG: Canada; BH: Republic of Ireland; BI: Republic of Singapore; BJ: Republic of Kenya; BK: Kingdom of Morocco.

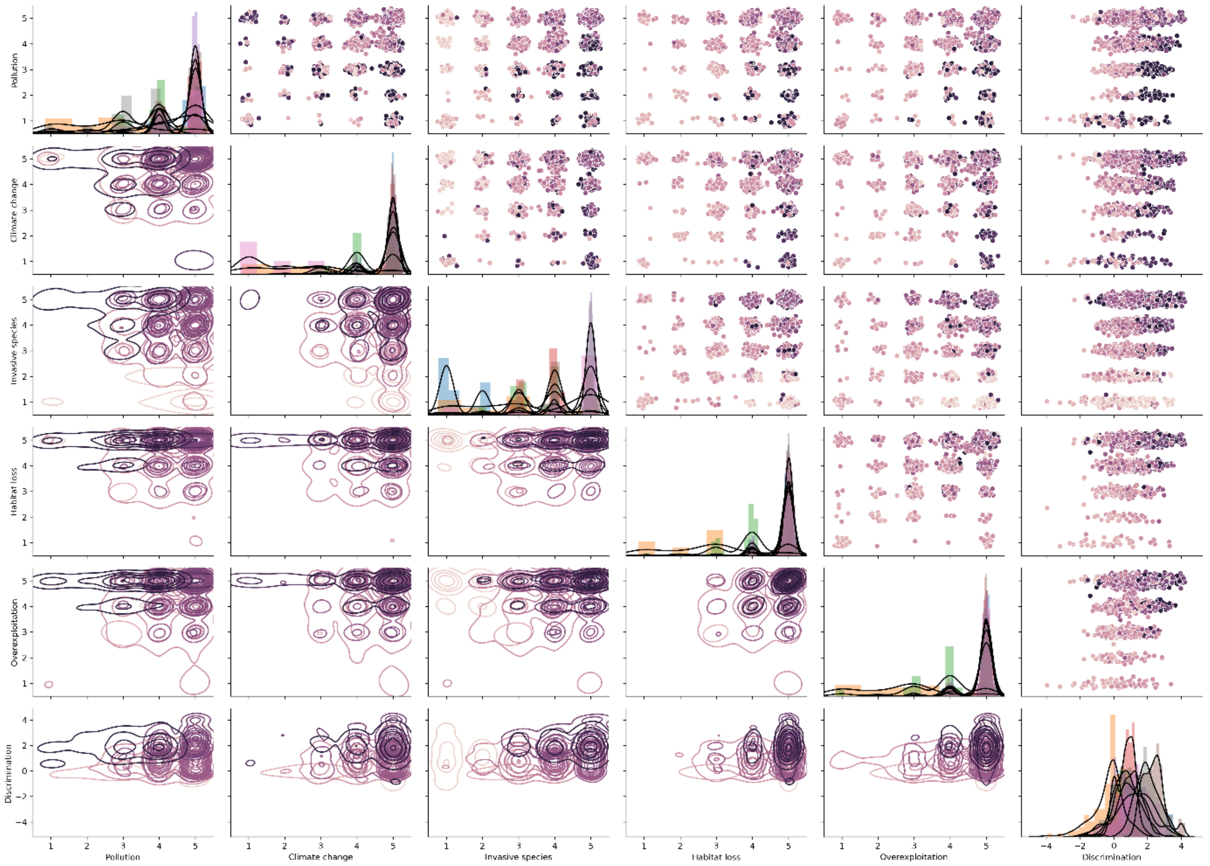

Supplementary Figure 3: This figure shows the relations between the different questionnaire items. It is a pairwise representation of the 6th dimensional feature value. The diagonal shows the frequency of the single values appearing in the questionnaires, colored by the eight clusters. Below the diagonal, KDE plots are used to display the joint distribution of the single questionnaire items per cluster. The same information is represented as scatterplots above the diagonal.

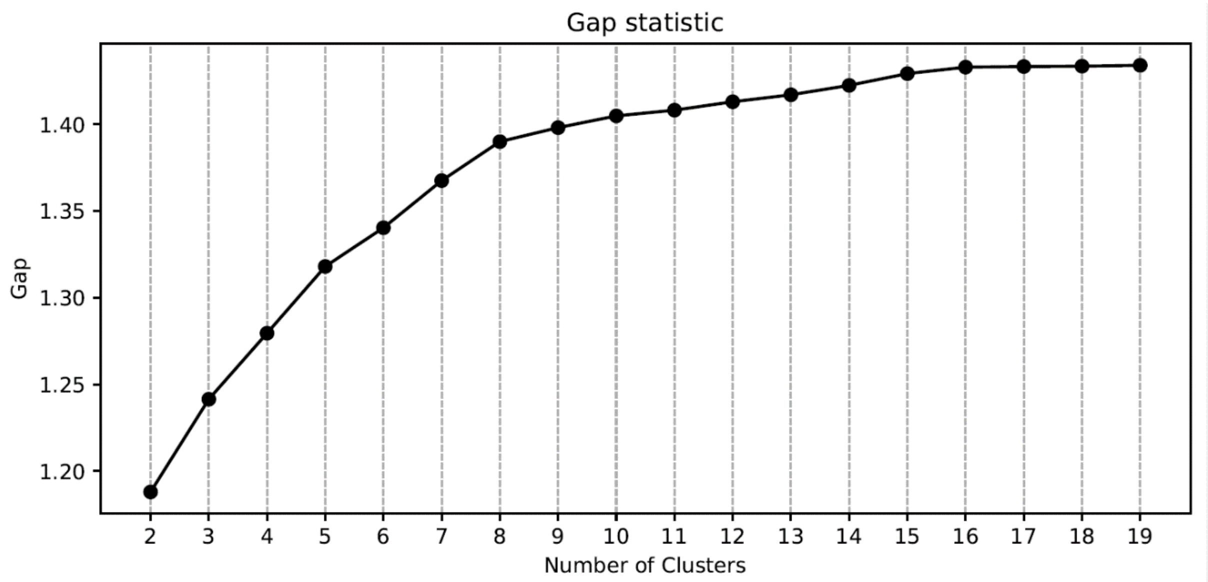

Supplementary Figure 4: Scree plot which shows the number of clusters against the gap statistic's gap value. The optimal number of clusters can be determined as 8.
